# High major histocompatibility complex class I polymorphism despite bottlenecks in wild and domesticated populations of the zebra finch (*Taeniopygia guttata*)

**DOI:** 10.1186/s12862-015-0546-3

**Published:** 2015-12-01

**Authors:** Daniel J. Newhouse, Christopher N. Balakrishnan

**Affiliations:** Howell Science Complex, East Carolina University, Greenville, NC 27858 USA

**Keywords:** Bottleneck, Immune, ND2, Positive selection, Genetic drift, Evolution

## Abstract

**Background:**

Two subspecies of zebra finch, *Taeniopygia guttata castanotis* and *T. g. guttata* are native to Australia and the Lesser Sunda Islands, respectively. The Australian subspecies has been domesticated and is now an important model system for research. Both the Lesser Sundan subspecies and domesticated Australian zebra finches have undergone population bottlenecks in their history, and previous analyses using neutral markers have reported reduced neutral genetic diversity in these populations. Here we characterize patterns of variation in the third exon of the highly variable major histocompatibility complex (MHC) class I α chain. As a benchmark for neutral divergence, we also report the first mitochondrial NADH dehydrogenase 2 (ND2) sequences in this important model system.

**Results:**

Despite natural and human-mediated population bottlenecks, we find that high MHC class I polymorphism persists across all populations. As expected, we find higher levels of nucleotide diversity in the MHC locus relative to neutral loci, and strong evidence of positive selection acting on important residues forming the peptide-binding region (PBR). Clear population differentiation of MHC allele frequencies is also evident, and this may be due to adaptation to new habitats and associated pathogens and/or genetic drift. Whereas the MHC Class I locus shows broad haplotype sharing across populations, ND2 is the first locus surveyed to date to show reciprocal monophyly of the two subspecies.

**Conclusions:**

Despite genetic bottlenecks and genetic drift, all surveyed zebra finch populations have maintained high MHC Class I diversity. The diversity at the MHC Class I locus in the Lesser Sundan subspecies contrasts sharply with the lack of diversity in previously examined neutral loci, and may thus be a result of selection acting to maintain polymorphism. Given uncertainty in historical population demography, however, it is difficult to rule out neutral processes in maintaining the observed diversity. The surveyed populations also differ in MHC Class I allele frequencies, and future studies are needed to assess whether these changes result in functional immune differences.

**Electronic supplementary material:**

The online version of this article (doi:10.1186/s12862-015-0546-3) contains supplementary material, which is available to authorized users.

## Background

Island colonizing species have long been an important target for evolutionary studies. The transition into novel ecological environments sets the stage for ecological adaptation, and sometimes, speciation [1]. On the other hand, the demographics of island colonization also pose challenges. For example, island colonization may often involve only a small number of founders, in turn resulting in a loss of genetic variation and possibly inbreeding. Therefore, island populations may show reduced heterozygosity and divergent allele frequencies relative to source populations [2, 3]. Over time, mating among this small pool of individuals may result in inbreeding depression, or reduced fitness from loss of genetic variation [4].

The loss in genetic variation may have especially profound effects on the immune system where genetic diversity is thought to be particularly important for certain loci [5]. Reduced immunogenetic variation has been shown in island populations of mammals [6], isolated populations of fish [7] and bottlenecked and island populations of birds [8–10]. Comparative studies of immune loci in island and mainland populations of birds, however, suggest that loss of immune function is not always the rule for island populations [11]. If population bottlenecks are relatively mild, short in duration and if selection favors polymorphism, diversity at immune loci may be maintained following colonization [12]. Furthermore, in many cases an increased investment in the innate response is apparent, perhaps mitigating the consequences of reduced diversity in adaptive immune loci [11].

A classic target of immune evolution studies has been the genes of the major histocompatibility complex (MHC) [13, 14]. The MHC contains the most variable genes known in vertebrates, with hundreds of alleles in some species [15, 16]. MHC molecules play a very important role in the adaptive immune response. These molecules bind and present peptides of degraded proteins on the surface of the cell to surveying T cells. If these presented peptides are non-self, as in the case of pathogen derived peptides, the T cell will elicit an immune response based on the type of MHC molecule it recognizes [17]. The high variability found in MHC molecules comes from the genes encoding the peptide-binding region (PBR), the site where peptides are loaded on to the molecule and presented on the cell surface. This variability is believed to be driven by strong positive selection, which is thought to broaden the spectrum of pathogens a population can protect themselves against [14, 18, 19]. Thus, the “evolutionary arms race” between MHC and pathogens likely drives the high variation seen in MHC loci [19].

Much of the early work characterizing the MHC [20, 21] and assessing its immunological role [22] in birds examined the domestic chicken (*Gallus gallus domesticus*). The chicken was described to have a “minimal essential” MHC containing a single dominantly expressed MHC Class I locus [23]. Recently, there have been a number of studies regarding MHC variation in non-model bird species, particularly in order Passeriformes [24–27]. Relative to chicken and other Galliforms, passerines typically have more MHC genes overall, as well as longer introns and pseudogenes [28–33], which Galliforms generally lack in the MHC. This greater complexity found in passerines could be driven by strong pathogen-mediated balancing selection [19].

Among passerines, the zebra finch (*Taeniopygia guttata*) has become an important model system for research in a diversity of fields, from ecology to neuroscience [34, 35] and was the second bird to have its genome sequenced [36]. Despite this, relatively little is known about patterns of genetic variation in zebra finch populations [37, 38]. Two subspecies of zebra finch are found throughout Australia (*T.g. castanotis*, hereafter “Australian”) and the Lesser Sunda islands of Southeast Asia (*T.g. guttata*, hereafter “Timor”). Mayr [39] originally hypothesized that the zebra finch colonized the Lesser Sunda Islands from Australia. More recently, with the support of 30 genetic markers, Balakrishnan & Edwards [37] showed that this colonization was associated with a severe genetic bottleneck that took place around one million years ago. The two subspecies differ phenotypically, with Timor finches being smaller and displaying reduced ornamentation [34]. Furthermore, these populations also differ at putatively neutral loci and differ significantly in the frequency of a large chromosomal inversion polymorphism [37, 40]. In addition to natural demographic changes in the wild, the zebra finch has also undergone multiple domestication events. Australian finches began the domestication process in the late 1800s [34] and have since become a popular species of household pet, as well as a model species for neurobiology and song learning research [41]. More recently, the Timor subspecies has been brought into captivity.

The zebra finch is also the only passerine in which efforts have been made to reconstruct the structure and organization of the entire MHC region [32]. Despite this, little is known about MHC polymorphism in the zebra finch. Previous work on the zebra finch MHC has demonstrated that there are numerous MHC Class II loci, but only a single expressed MHC Class I locus [32, 42]. In this study, we assess the role of historical bottlenecks on the variability of the functionally important MHC class I PBR in Australian, Timor and North American domesticated populations of zebra finches. We also provide the first analysis of variation in the mitochondrial NADH dehydrogenase 2 (ND2) in the zebra finch as a comparative neutral marker. In doing so, we not only continue the characterization of neutral genetic differences between the two wild zebra finch populations [37], but also characterize potential differences in functional loci between the wild and domesticated populations that play such an important role in biological research.

## Methods

### DNA samples

Twenty-nine DNA samples from wild Australian (*T.g. castanotis*) birds were used in this study. Sixteen of these were previously analyzed by Balakrishnan & Edwards [37] with field collection and permits described therein. As in the previous study [37], a subset of the samples from Australia (*n* = 13) were provided as purified DNA by Dave Runciman (formerly of LaTrobe University). Tissue samples from Balakrishnan and Edwards [37] are accessioned at the Harvard University Museum of Comparative Zoology and the Academy of Natural Sciences at Drexel University. These wild Australian birds were sampled at nine locations throughout the continent: Queensland, Selwyn Range (21° 17′ S, 140° 27′ E), Longreach (17° 59 S, 138° 50′ E, *n* = 8), Brixton (23° 32′, 144° 57′); Northern Territory, Alice Springs (23° 42′ S 133° 52′ E); Western Australia, Fitzroy Crossing (18° 11′, 125 36′), Hamersley Range (21° 53′ S, 116° 24′ E), Kununurra, (15° 51′ S, 128° 44′ E); Victoria, Wunghnu, (36° 9′ S, 145 26′ E). We also used 12 DNA samples from wild Timor (*T.g.guttata*) birds sampled by Runciman on the islands of West Timor (*n* = 6) and Lombok (*n* = 6). We used three additional domesticated Timor samples from the captive colony at the University of Illinois at Urbana-Champaign (courtesy of David Clayton). Due to the challenging logistics of fieldwork in the Lesser Sundas, and hence, small sample size, we lump wild and domesticated birds together for this subspecies.

We also sampled domesticated Australian zebra finches from four captive colonies in the United States. We used four samples from the East Carolina University (ECU) colony, two samples from the University of Illinois at Urbana-Champaign (courtesy of David Clayton), 11 samples from the University of Chicago (courtesy of Sarah London) and 15 samples from the USGS National Wildlife Health Center in Madison, WI (courtesy of Erik Hofmeister). The samples (*n* = 4) collected at ECU were approved by the East Carolina University Institutional Animal Care and Use Committee (IACUC) under protocol D285. These four birds were sampled as part of ongoing neurogenomic research under protocol D285 and were euthanized via decapitation. Tissues were snap frozen on dry ice and DNA was extracted using Qiagen DNeasy kits following manufacturer’s protocols. Additional file 1 provides information regarding sample location and locus genotyped for each sample. Seventy-one samples were sequenced for MHC class I and 59 of 71 samples used in the MHC analysis were also sequenced for ND2 along with five additional samples. Not all samples were genotyped at both loci, in part to due to limited DNA availability for some samples.

### MHC class I genotyping

Previous studies utilizing BAC libraries, southern blot [32] and RNAseq [42] revealed that only a single MHC class I locus is expressed in zebra finches. Thus, we amplified a 325 bp section of exon 3 of the MHC class I α chain using polymerase chain reaction (PCR). For each PCR reaction, we combined 1.00 μl of genomic DNA with 16.25 μl molecular grade ddH2O, 2.50 μl each of 10x MgCl_2_ buffer and dNTPs, 1.25 μl each of 10 μM MHC 1.3 forward (5′-ATGGGTCTCTGTGGGTACAATC-3′) and reverse (5′-CCCACAGGAATTACCATGTTCC-3′) primers, and 0.25 μl Taq DNA polymerase. Primers were designed to flank exon 3 based on the zebra finch genome assembly (taeGut1) [36] and the target regions are highly conserved in Passerines [32]. Each reaction took place under the following PCR conditions: 3 min at 95 °C for denaturation, then 34 cycles of 20 s at 95 °C, 20 s at 57 °C and 45 s at 72 °C.

Following PCR, we separated products on a 1 % agarose gel to ensure proper amplification of the DNA during PCR. We then purified PCR products using an Epoch GenCatch PCR Extraction Kit (Epoch Life Sciences) and prepared for Big Dye terminator sequencing reaction. Each Big Dye reaction contained 2.00 μl of 1 μM MHC 1.3 forward or reverse primer, 4.50 μl 5x extender buffer, 1.00 μl Big Dye, 7 μl H20 and 5.50 μl purified DNA. This 20 μl reaction underwent the following protocol: 1 min at 96 °C, then 26 cycles of 10 s at 96 °C, 5 s at 50 °C and 4 min at 60 °C. We then purified Big Dye reaction products using sephadex columns and sequenced using an ABI 3130 at the ECU Genomics Core Facility.

### ND2 genotyping

Mitochondrial genes are useful markers in reconstructing population histories and comparing patterns of divergence in markers experiencing high levels of selection, such as the MHC [14]. Thus, we sequenced a portion of the mitochondrial ND2 gene to provide a complementary perspective to contrast with our MHC class I analysis as well as to compare with mitochondrial studies in other taxa. Following the same protocol listed above, altering only the PCR annealing temperature to 55 °C, we genotyped a 550 bp region of the mitochondrial ND2 gene using the forward L5216 (5′-GGCCCATACCCCGRAAATG-3′) and reverse H5766 (5′-RGAKGAGAARGCYAGGATYTTKCG-3′) primers [43].

### DNA sequence analysis

We used *Geneious* v6.0.5 (Biomatters) to view sequence data, assemble and align sequences, remove primers, call polymorphic sites and perform consensus sequence alignment for downstream analyses. After trimming, we obtained a 250 bp MHC class I and 483 bp ND2 consensus sequence for each individual. We determined both MHC haplotypes using the *PHASE* software [44, 45] implemented in *DnaSP* v5 [46], which reconstructs haplotypes based on population genetic data. Measures of nucleotide diversity (π), synonymous (π_s_) and nonsynonymous (π_a_) nucleotide diversity, haplotype diversity (HD) [47], 95 % HD confidence interval by coalescent simulation [48] and population differentiation (K_ST_) [49] were also calculated in *DnaSP* v5. We chose to estimate K_ST_ [49] rather than F_ST_, because K_ST_ is particularly suitable for smaller sample sizes, and for loci with high polymorphism, such as the MHC [50]. We used our haplotypes to construct a network in the program *PopART* [51] to show relationships among the individuals sampled from different populations. The TCS algorithm was selected to make this network based on its implementation of statistical parsimony [52].

We identified signatures of positive selection in our MHC class I sequences with the program *omegaMap* [53] by calculating ω, the ratio of non-synonymous substitutions (d_N_) to synonymous substitutions (d_S_), where ω (d_N_/d_S_) > 1 indicates positive selection. *OmegaMap* requires prior distributions for each parameter and we selected, as suggested by the manual, the following for ω: omega prior = inverse (0.010, 100) and omega model = independent, which allows a unique ω value for each codon. We ran the simulation twice for 500,000 generations and combined each run with the suggested burn in of 50,000 (10 %) iterations using the *Summarize* module provided by the program. We visualized results using *R* v3.0.2. Using the homology based approach implemented by Promerová et al. [54] based on Wallny et al. [55] and Koch et al. [56], putative PBR sites were determined by aligning GenBank sequences from the duck (*Anas platyrhynchos*) MHC [GenBank: AY2994416.1] [57] and scarlet rosefinch (*Carpodacus erythrinus*) MHC class I exon 3 [GenBank: FJ392790] [54] with these zebra finch sequences. We were then able to determine whether sites showing signs of positive selection correspond to putative peptide binding residues.

### Bottleneck simulations

To test whether patterns of variation could be explained by genetic drift acting on our MHC class I sequences during colonization of the Lesser Sunda Islands, we ran bottleneck simulations in *BottleSim* v2.6 [58]. We used the observed MHC allele frequencies in the Australian subspecies to examine reductions of allelic diversity under neutrality in bottlenecks of varying severity. We performed simulations using the following parameters: average lifespan = 2 years, age to sexual maturity = 1 year, generation overlap = 50 % and the observed Australian MHC class I allele frequencies. We varied rates of population growth post-bottleneck to see how the bottleneck affected allelic diversity during island colonization. Each simulation ran for 1,000 iterations with a bottleneck that reduced populations to either 10 or 100 individuals, from an initial population size of 20,000. A bottleneck event with 10–100 founders is consistent with previous estimates of colonization of the Lesser Sunda Islands [37]. Post bottleneck populations were allowed to grow exponentially at 10, 20 and 50 % for 34–100 years. Simulations of short duration (e.g. 34 years) were run in some cases because population sizes became too large, making the simulation too computationally intensive.

## Results

### MHC polymorphism & divergence

**Fig. 1:**
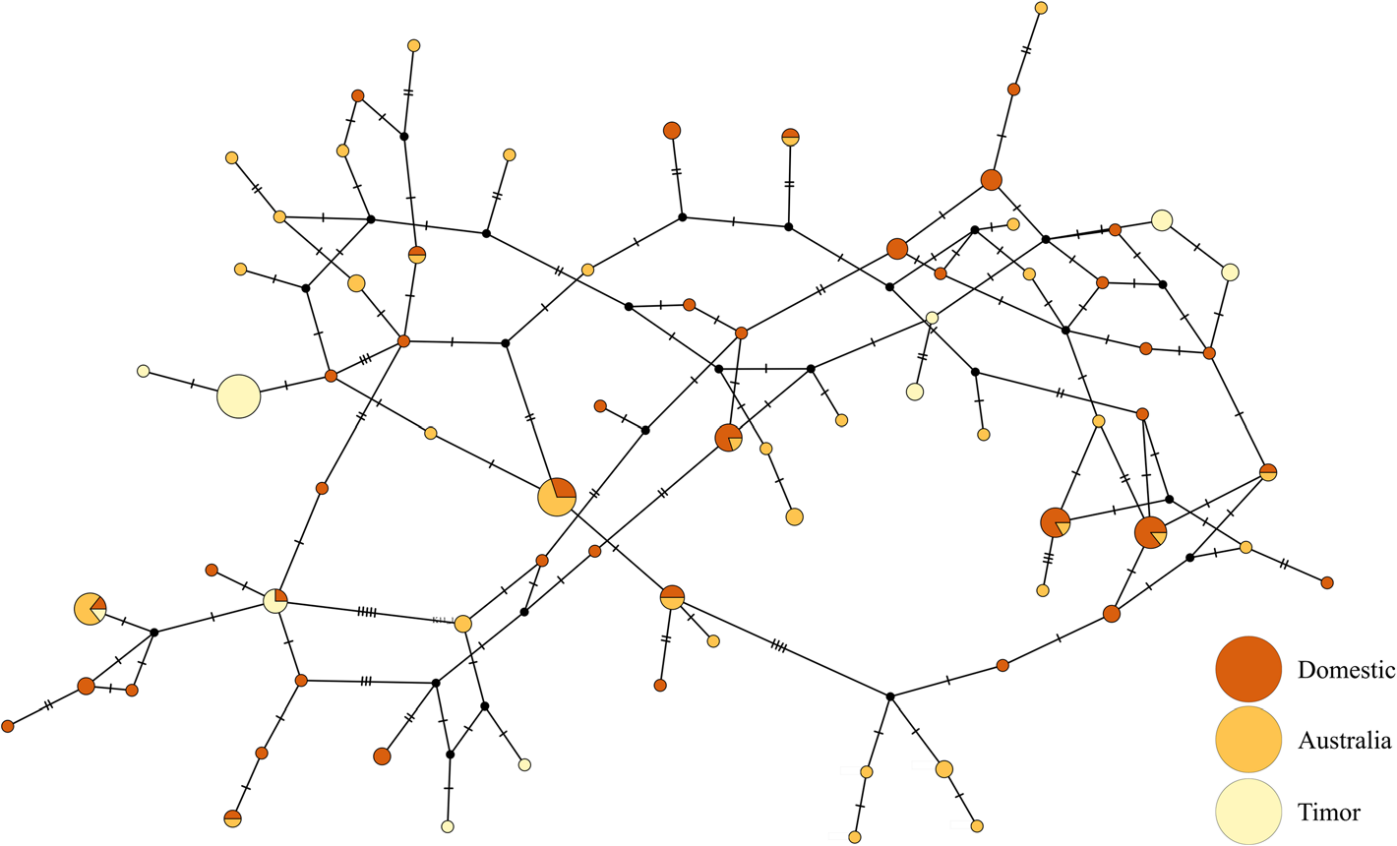
Parsimony network created in *PopART* showing relationships between the different MHC alleles and populations. The size of the nodes is proportional to the number of individuals with that particular allele. Coloration of the nodes represents the frequency of each allele among the study populations. Black circles represent missing alleles

**Fig. 2:**
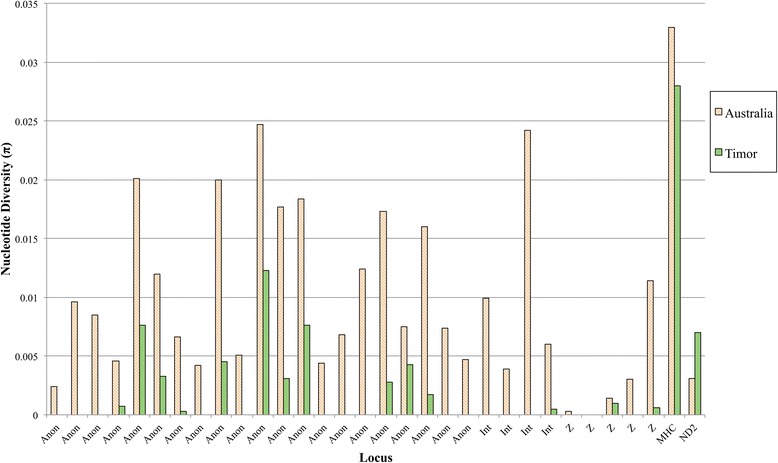
Island and mainland nucleotide diversity (π) plotted next to each other for all reported loci. Anonymous loci (Anon), introns (Int) and Z linked loci (Z) reported by Balakrishnan & Edwards [37] as well as MHC class I and ND2 from the present study

We successfully sequenced a total of 25 Australian, 14 Timor and 32 Domestic zebra finches for exon 3 of the MHC class I gene [GenBank: KT595736-KT595877]. Using the *PHASE* software [44, 45] implemented in *DnaSP* v5 [46], 74 unique nucleotide and amino acid haplotypes were reconstructed among the three populations (Table 1, Fig. 1 and Additional file 2). Within each of these populations, nucleotide diversity (π) is relatively high: Timor = 0.028, Wild Australia = 0.033, Domesticated = 0.033 (Table 1 and Fig. 2). We also observe a similar pattern of synonymous (π_s_) and nonsynonymous (π_a_) site nucleotide diversity (Table 1). Within the Timor population, π_a_ is still high (π_a_ = 0.033), indicating maintenance of nonsynonymous diversity (Table 1). This is also reflected in the amino acid sequence alignment (Additional file 2). Similarly, haplotype diversity (HD) is high in each of the three sampled populations, with the Timor population exhibiting the lowest diversity (Table 1). Despite this, Timor MHC haplotype diversity is higher than 29 out of 30 (96 %) of previously reported neutral loci (Additional file 3). Additionally, the lower limit of the 95 % confidence interval, as estimated by coalescent simulation in *DNAsp*, exceeds HD in 28/30 (93 %) of the neutral loci sampled in Timor birds (HD_lower_ = 0.693, Additional file 3).

**Table 1:** Summary statistics for the three zebra finch populations

	H/N	HD	H/N	HD	π	π_s_	π_a_	π	π	ω
	(MHC)	(MHC)	(ND2)	(ND2)	(MHC)	(MHC)	(MHC)	(ND2)	(Neutral)	(MHC)
Domestic - USA	41/64	0.978	7/27	0.735	0.033	0.011	0.040	0.006	n/a	5.61
(*T. g. castanotis*)										
Australian	35/50	0.971	12/20	0.811	0.033	0.010	0.040	0.003	0.011	5.57
(*T. g. castanotis*)										
Timor	10/28	0.772	3/12	0.530	0.028	0.011	0.033	0.007	0.002	5.25
(*T. g. guttata*)										
Overall	74/142	0.978	22/59	0.692	0.032	0.011	0.041	0.006	n/a	5.48

Number of unique haplotypes (H) for the total number of individuals sampled (N), haplotype diversity (HD), overall nucleotide diversity (π), synonymous (π_s_) and nonsynonymous (π_a_) nucleotide diversity, π for neutral loci reported by Balakrishnan & Edwards [37] and average MHC class I ω reported here

Although each population exhibits high overall levels of nucleotide and haplotype diversity, there is a clear pattern of population divergence (Table 2). As expected, pairwise K_ST_ values reveal that the highest degree of divergence is between wild Australian and Timor zebra finch subspecies, which diverged 1–2 million years ago (K_ST_ = 0.073, p < 0.001). Domesticated and wild Australian populations showed the lowest levels of divergence, but the two populations were nevertheless significantly differentiated (K_ST_ = 0.016, p = 0.020). These patterns can also be observed in the TCS network (Fig. 1), as Australian zebra finches share fewer alleles with the Timor subspecies relative to domesticated birds.

**Table 2:** Pairwise K_ST_ values showing genetic divergence between populations with values previously reported

Comparison	K_ST_	*p*-value of K_ST_	K_ST_	*p*-value of K_ST_	Neutral markers
	(MHC)		(ND2)		
Domestic vs. Australian	0.016	0.020	0.050	0.011	0.062 (F_ST_)^a^
Australian vs. Timor	0.073	<0.001	0.665	<0.001	0.197^b^

^a^[38]

^b^[37]

Summary statistics describing polymorphism and diversity are also reflected in the TCS [50] based parsimony network (Fig. 1). The high haplotype diversity is emphasized by the fact that most haplotypes are only carried by a small number of individuals (0.521 haplotypes per individual). The significant genetic divergence between Timor and Australian finches is reflected in the limited haplotype sharing among subspecies (Fig. 1). Only two haplotypes were shared among subspecies, and the subspecies are therefore not reciprocally monophyletic for MHC Class I alleles. Also of note is that Timor alleles are dispersed across multiple parts of the network, rather than clustering together in one area.

We found broad overlap between alleles carried by domestic and wild Australian zebra finches (10 shared haplotypes). There are however, numerous (25) alleles found in wild population but not represented in our captive sample. Likewise, 31 alleles found in captive birds were not detected in wild-derived samples. This is further reflected by the large number of unique amino acid sequences found in each population as well as the significant genetic differentiation among captive and wild birds (Additional file 2 and Table 2).

### ND2 polymorphism & divergence

**Fig. 3:**
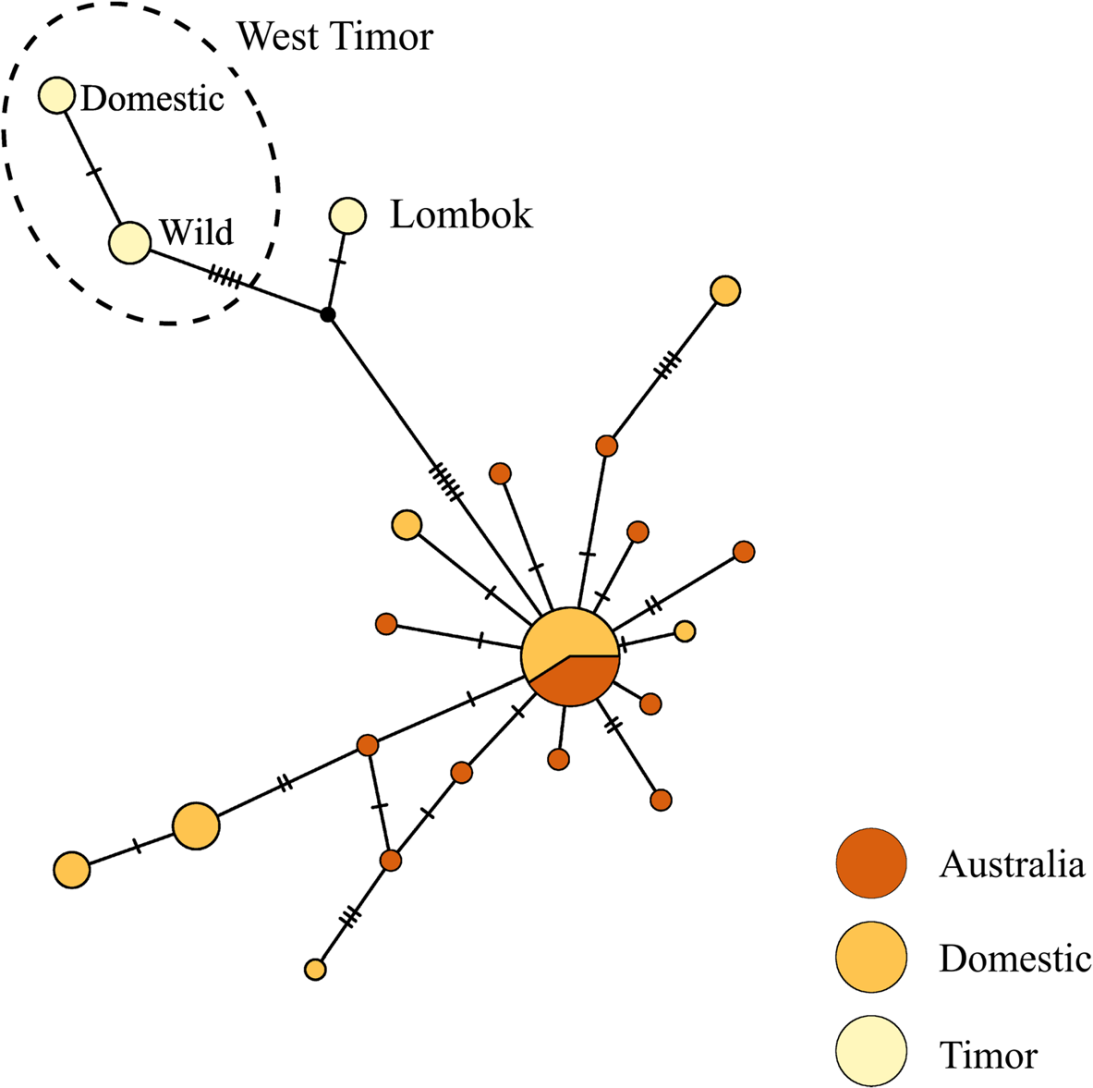
Parsimony network created in *PopART* showing relationships between the different ND2 alleles and populations. The size of the nodes is proportional to the number of individuals with that particular allele. Coloration of the nodes represents the frequency of each allele among the study populations. Black circles represent missing alleles. Timor individuals are represented by sampling location

We successfully sequenced 20 Australian, 27 Domestic and 12 Timor individuals for a 483 bp region of the ND2 mitochondrial gene [GenBank: KT595878-KT595935]. Compared with the MHC, as expected, ND2 nucleotide diversity (π) is relatively low: Australia = 0.003, Domestic = 0.006 and Timor = 0.007 (Table 1). Although the Timor population exhibits the greatest ND2 nucleotide diversity, Timor ND2 HD is the lowest (Table 1). Pairwise K_ST_ values show significant divergence between wild Australia and Timor allele frequencies (K_ST_ = 0.665, p < 0.001) and slight, but statistically significant, divergence between wild and domesticated populations (K_ST_ = 0.050, p = 0.011). The relatively low levels of diversity and significant population divergence are reflected in the large number of shared alleles between Australian and Domestic samples and separation of Australian and Timor samples in our ND2 haplotype network (Fig. 3). Furthermore, the Timor samples further segregate into two distinct haplotype groups based on sampling locality (West Timor and Lombok islands). Seven mutational steps separate these two clusters.

### Tests of selection of MHC nucleotides

**Fig. 4:**
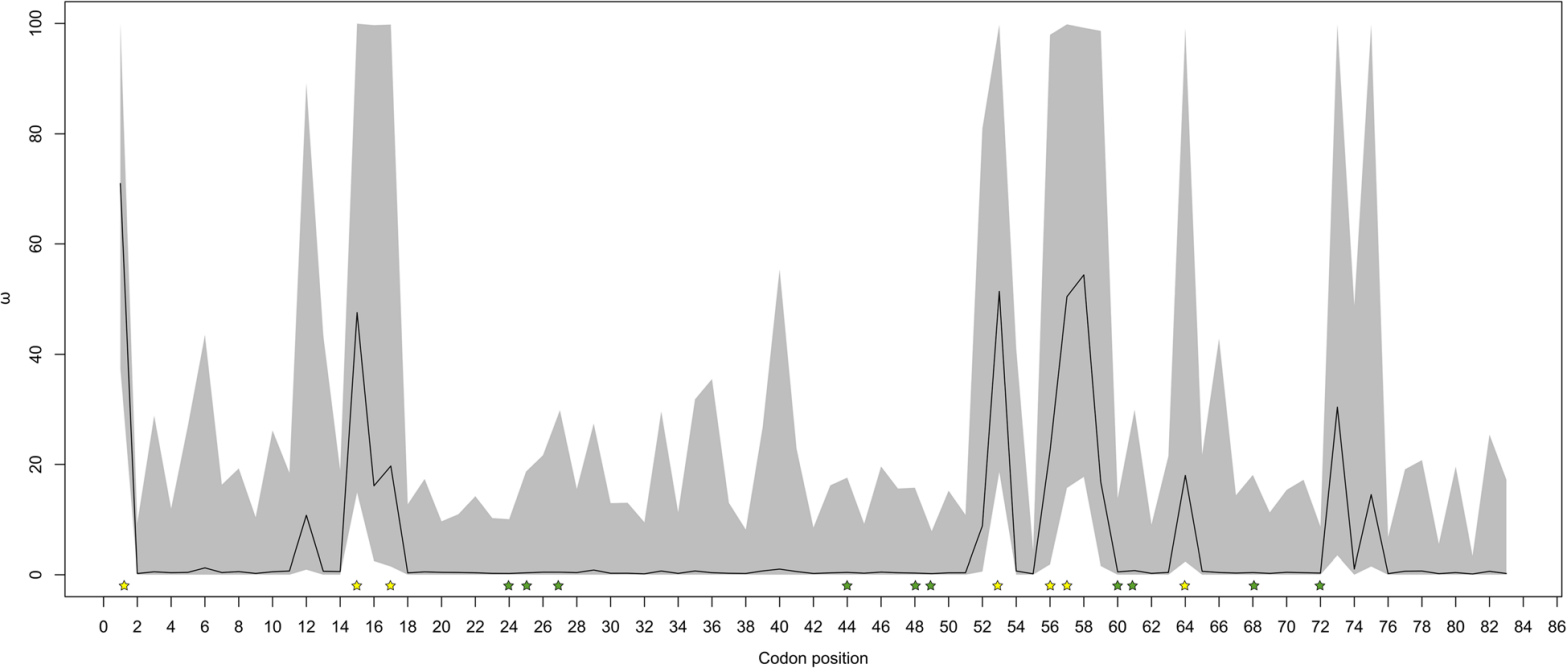
Output from *omegaMap* plotting ω (d_N_/d_S_) against codon position for the wild Australian population. The black lines represent omega and the grey shading represents 95 % highest posterior probability densities (HPD). Stars represent sites encoding the PBR, with yellow stars indicating ω > 1 and green stars indicating ω < 1

Selection analyses using *omegaMap* [53] revealed strong evidence for positive selection on exon 3 of the MHC class I locus. The average value of ω averaged across all codon sites is much higher than one; the expectation for neutrality (Table 1, ω = 5.48), and 17–22 sites show signatures of positive selection with significant posterior probabilities in each population (Fig. 4 and Additional files 4, 5, 6, 7 and 8). Alignment of our sequences with those of previously reported MHC class I of the duck (*Anas platyrhynchos*) and scarlet rosefinch (*Carpodacus erythrinus*) allowed us to determine which of our sites were putative residues of the PBR and whether these sites exhibited signatures of positive selection. Our sequences contained 17 putative PBR residues, seven of which exhibited strong signatures of positive selection in each population (Fig. 4 and Additional files 5 and 7).

### Neutral expectations following population bottlenecks

**Fig. 5:**
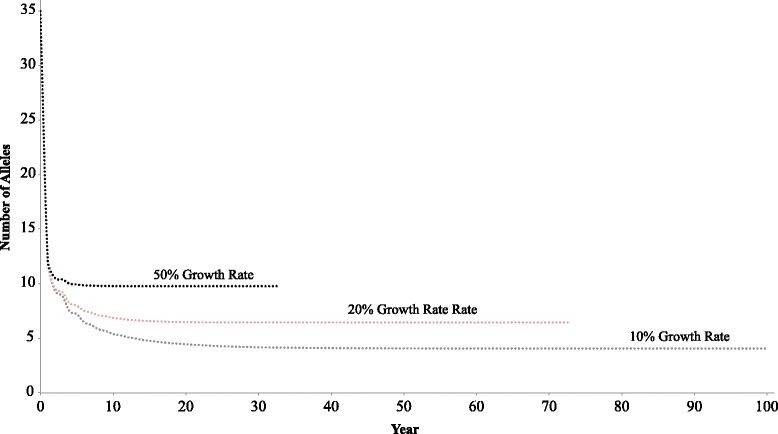
*BottleSim* output with 10, 20 and 50 % growth rate post-bottleneck of 10 individuals

Our bottleneck simulations revealed that based on drift alone, the Timor zebra finch should carry between four and 10 MHC alleles given a founding population of 10 individuals, and over 21 alleles given a founding population of 100 individuals (Fig. 5 and Additional file 9). Under a predicted scenario based on previous coalescent-based estimates [37], in which a dramatic bottleneck down to 10 individuals was followed by exponential growth rate of 10 %, we observed a reduction from 35 initial alleles on the mainland to 4.06 (±0.034) alleles after island colonization. Increasing the growth rate to 20 % post-bottleneck reduced the number of alleles from 35 to 6.45 (±0.043). Lastly, a 50 % exponential growth rate following the founder event reduced the number of alleles to 9.78 (±0.053), very similar to our observed value of 10 alleles. Under a less severe bottleneck scenario of 100 individuals, simulated allelic diversity estimates far exceeded our observed values (Additional file 9).

## Discussion

### Polymorphism maintenance in the MHC

Population bottlenecks are expected to result in reduced neutral genetic variation [2]. This pattern has previously been shown in both insular and domesticated zebra finch populations [37, 38]. Of 30 nuclear loci, including intronic regions, sex-linked and anonymous loci, Balakrishnan & Edwards [37] found over half (16 loci) to be invariant in Timor zebra finches. On average, Timor zebra finches exhibit a 75 % drop in nucleotide diversity of neutral markers. We have shown here that even in the presence of this genome wide reduction in variation, Timor zebra finches have maintained a high level of diversity in the functionally important MHC (Fig. 2 and Additional file 3). The MHC class I locus sequenced here thus stands in marked contrast to previously sampled putatively neutral loci in zebra finches (Table 1).

One possible explanation for the elevated MHC diversity relative to neutral markers is if selection has acted to maintain polymorphism. A few of lines of evidence support this conclusion. First, we find strong evidence of selection acting on this MHC locus, as evidenced by elevated d_N_/d_S_ values (Table 1) [59]. Second, the observed nucleotide diversity of the MHC locus in Timor finches is outside the range of nucleotide diversity observed in previously sequenced neutral loci [37]. Even though loci that are highly polymorphic in the Australian subspecies tend to be more polymorphic in the Timor subspecies, the MHC locus has maintained more nucleotide diversity than all putatively neutral, autosomal loci (Fig. 2). Further, our bottleneck simulations revealed that under many scenarios, including those that align with previous estimates of population demography [37], neutral divergence would result in a more severe reduction in MHC diversity than we observed [Table 1 and Fig. 5].

Given the unique biology of the MHC and the uncertainty of zebra finch historical demography, completely ruling out neutral explanations for the observed high level of MHC diversity, however, is challenging. Under some demographic scenarios, neutral expectations based on simulations closely approximate observed patterns of MHC diversity. For example, if the bottleneck associated with island colonization was less severe than previously estimated [37], or if population growth were relatively fast following island colonization, higher levels of MHC diversity would be expected [12]. Indeed, we obtain higher than observed levels of post-bottleneck MHC allelic diversity under a less severe bottleneck scenario suggesting that drift alone could maintain allelic diversity (100 individuals, Additional file 9). Previous, estimates suggest that a small number of individuals (~10) likely colonized the islands. Confidence intervals around this estimate, however, are broad (95 % CI, ≈ 9–9000 individuals), making colonization of the islands by over 100 founders possible.

The process of domestication is also expected to reduce genetic diversity, particularly if populations are founded using relatively small numbers of individuals from the wild, or if selective breeding has been strong. Forstmeier et al. [38] found domesticated zebra finches to have slightly reduced genetic variation at 12 microsatellite loci and significant population differentiation when compared with wild zebra finches. However, Forstmeier et al. also found that a relatively high level of neutral genetic polymorphism has been maintained through domestication [38]. We find the same pattern in the MHC class I (Table 1), albeit with a lower degree of differentiation (Table 2). The domestic birds utilized in this study were domesticated relatively recently, breed randomly (i.e. are not selectively bred) and encounter pathogens found in captivity. Therefore, it is not surprising to see high levels of MHC class I sequence and haplotype diversity as seen in other domesticated vertebrates [60]. We found 31 alleles in domesticated birds not found in the wild. These novel alleles may indicate adaptation to the pathogen community found in captivity (Fig. 1), although further sampling along with functional testing is needed for verification.

High diversity in the MHC suggests positive selection and indeed, there are abundant nonsynonymous polymorphisms throughout these Class I sequences (Table 1 and Additional file 2). Our sequences contained 17 putative PBR sites, seven of which exhibited strong evidence of positive selection in each population (Fig. 4 and Additional files 4, 5, 6, 7 and 8). The sites we report to be under positive selection have also been previously reported in another passerine, the scarlet rosefinch (*Carpodacus erythrinus*), that displays high levels of MHC class I variation [54]. We also observe several sites experiencing positive selection outside of the putative PBR, potentially revealing additional sites important in peptide binding. As most reported estimates of MHC nucleotide diversity are from species with multiple expressed MHC loci [26], direct comparisons of diversity among birds, in particular among passerines, are complicated by inter-species variation in MHC copy number.

The MHC plays a vital role in antigen presentation to the immune system, so selection is expected to maintain diversity to broaden pathogen recognition capability [14]. Although there have been a number of studies correlating bottlenecked populations with reduction in MHC variation [6, 10, 61], there have also been several studies showing the opposite, as balancing selection appears to counteract drift [12, 60, 62, 63]. The specific details of population demography play a critical role in determining the outcome following colonization [64]. Recent work has revealed that balancing selection mediated either by negative frequency dependent selection or heterozygote advantage can actually decrease rather than maintain MHC polymorphism after a bottleneck [64, 65]. Given sufficient time and recovery after a bottleneck, however, balancing selection will again act to maintain polymorphism [64]. Likewise, gene conversion has also been shown to maintain MHC variation [66, 67]. Lastly, differential pathogen pressures faced in each of these populations may contribute to the high diversity and divergence among populations [18].

### Divergence between Australian, island and domesticated populations

The two zebra finch subspecies appear to have been isolated since the early Pleistocene or late Pliocene [37]. Thus, our estimates of genetic differentiation in the MHC class I and ND2 are consistent with the current understanding of zebra finch population history [34]. This prolonged history of isolation of subspecies is also reflected in MHC Class I haplotype diversity, as only two of 74 MHC Class I haplotypes are shared between subspecies (Fig. 1; K_ST_ = 0.073). Network analysis of MHC Class I alleles shows that the Timor alleles are distributed across multiple parts of the network, indicating that multiple colonizers carrying divergent alleles founded the island population. K_ST_ estimates between subspecies for MHC Class I are lower than from neutral markers. This pattern, however, is expected, as measures of population divergence are necessarily lower for highly polymorphic markers [68]. Balancing selection could also contribute to this reduced K_ST_ [69].

To provide a more complete picture of population genetic variation in this model system, we sequenced a portion of the mitochondrial ND2 gene. Mitochondrial genes have long been used as markers of population history [70], and thus provide a point of contrast for loci under selection like the MHC [14]. Despite the utility of mitochondrial markers as a benchmark for taxonomic delineation and history, there has not previously been a mitochondrial DNA (mtDNA) survey in zebra finches. We found that zebra finch subspecies were reciprocally monophyletic for mtDNA alleles (Table 1 and Fig. 3), making this the first genetic marker to show monophyly of zebra finch subspecies [71]. Accordingly, there is highly significant population divergence between the subspecies (K_ST_ = 0.665, p < 0.001, Table 2).

ND2 polymorphism in zebra finches is comparable to previous reports of ND2 nucleotide diversity in other passerines (π = 0.003–0.007, Table 1) [72–74]. Interestingly, ND2 nucleotide variation is twice as high in the Timor subspecies (π = 0.007) as in the Australian subspecies (π = 0.003) (Table 1). This high nucleotide diversity on the islands is a consequence of the existence of two divergent mtDNA haplotype groups (separated by seven mutational steps) in our sample of 12 birds. Each node in our Timor network represents a distinct sampling locality (West Timor, Lombok, and Captive) with the captive samples separated by one mutation from the wild Timor samples. The high divergence between samples from West Timor and Lombok islands suggests long-term isolation of those populations, but more detailed sampling throughout the Timor subspecies range is still needed to formally test this.

Zebra finch populations were domesticated in the 1800s. As a result, domestic and wild populations have relatively low, but statistically significant, divergence in MHC Class I allele frequencies (K_ST_ = 0.016). These two populations also show statistically significant differences in ND2 allele frequencies (K_ST_ = 0.050). Together these finding provide evidence of genetic distinctiveness of wild populations in Australia and domesticated populations in the USA.

## Conclusions

Domestication and artificial selection have important and predictable consequences [75–77]. However, we find high polymorphism despite a bottleneck in the colonization of the Lesser Sunda islands, and like Forstmeier et al. [38], we find high levels of nucleotide diversity in captive zebra finches as well. We also found significant differences in MHC allele frequencies between zebra finch subspecies and domesticated populations, indicating potential immune adaptation to the pathogen pressures faced by each population [59] or the impact of genetic drift. Although the discordance between neutral and MHC polymorphism is striking, improved resolution of historical demography is required in order to determine whether this disparity is driven by selection. Zebra finches are a model system in a diversity of fields. Genetic divergence in functional loci, such as that shown here for the MHC, may have important implications for research in the fields for which zebra finch is a model system. For example there is already evidence that domesticated and wild zebra finches differ in aspects of social behavior [78]. Comparisons of these populations in terms immunological or gene expression responses to immune challenges will provide insight into the functional consequences of the MHC class I variability we report here.

## Availability of supporting data

All sequences used in this study have been deposited in GenBank under accession numbers KT595736-KT595877 for MHC class I and KT595878-KT595935 for ND2.

## Additional files

Additional file 1:
**Sample name, origin and locus genotyped for each sample used in this study.** (XLS 33 kb) Additional file 2:
**Amino acid alignment of unique MHC class I sequences from each population.** For each unique allele, the first number represents allele ID and the second number represents the total number of individuals in the population carrying the allele. (PNG 1776 kb) Additional file 3:
**Island and mainland haplotype diversity (HD) plotted next to each other for all reported loci.** Anonymous loci (Anon), introns (Int) and Z linked loci (Z) reported by Balakrishnan & Edwards [37] as well as MHC class I and ND2 from the present study. (PNG 1820 kb) Additional file 4:
**Output from** ***omegaMap*** **plotting posterior probability of positive selection against codon position for the wild Australian population.** Stars represent sites encoding the PBR, with yellow stars indicating ω > 1 and green stars indicating ω < 1. (PNG 773 kb) Additional file 5:
**Output from** ***omegaMap*** **plotting ω against codon position for the domesticated population.** The black lines represent omega and the grey shading represents 95 % highest posterior probability densities (HPD). Stars represent sites encoding the PBR, with yellow stars indicating ω > 1 and green stars indicating ω < 1. (PNG 1028 kb) Additional file 6:
**Output from** ***omegaMap*** **plotting posterior probability of positive selection against codon position for the domesticated population.** Stars represent sites encoding the PBR, with yellow stars indicating ω > 1 and green stars indicating ω < 1. (PNG 809 kb) Additional file 7:
**Output from** ***omegaMap*** **plotting ω against codon position for the Timor population.** The black lines represent omega and the grey shading represents 95 % highest posterior probability densities (HPD). Stars represent sites encoding the PBR, with yellow stars indicating ω > 1 and green stars indicating ω < 1. (PNG 1064 kb) Additional file 8:
**Output from** ***omegaMap*** **plotting posterior probability of positive selection against codon position for the Timor population.** Stars represent sites encoding the PBR, with yellow stars indicating ω > 1 and green stars indicating ω < 1. (PNG 870 kb) Additional file 9:
***BottleSim*** **output showing the final number of alleles (±SE) at 10, 20 and 50 % post-bottleneck growth rate for a bottleneck size of 10 and 100 individuals.** (PDF 41 kb)

## Abbreviations

MHC: Major histocompatability complex
ND2: NADH dehydrogenase 2
PBR: Peptide-binding region
HD: Haplotype diversity
mtDNA: Mitochondrial DNA
