# Supplementary figures and images for: High major histocompatibility complex class I polymorphism despite bottlenecks in wild and domesticated populations of the zebra finch (Taeniopygia guttata)
Source: BMC Evol Biol. 2015 Dec 1;15:265. doi: 10.1186/s12862-015-0546-3 (PMC4667478; doi:10.1186/s12862-015-0546-3)

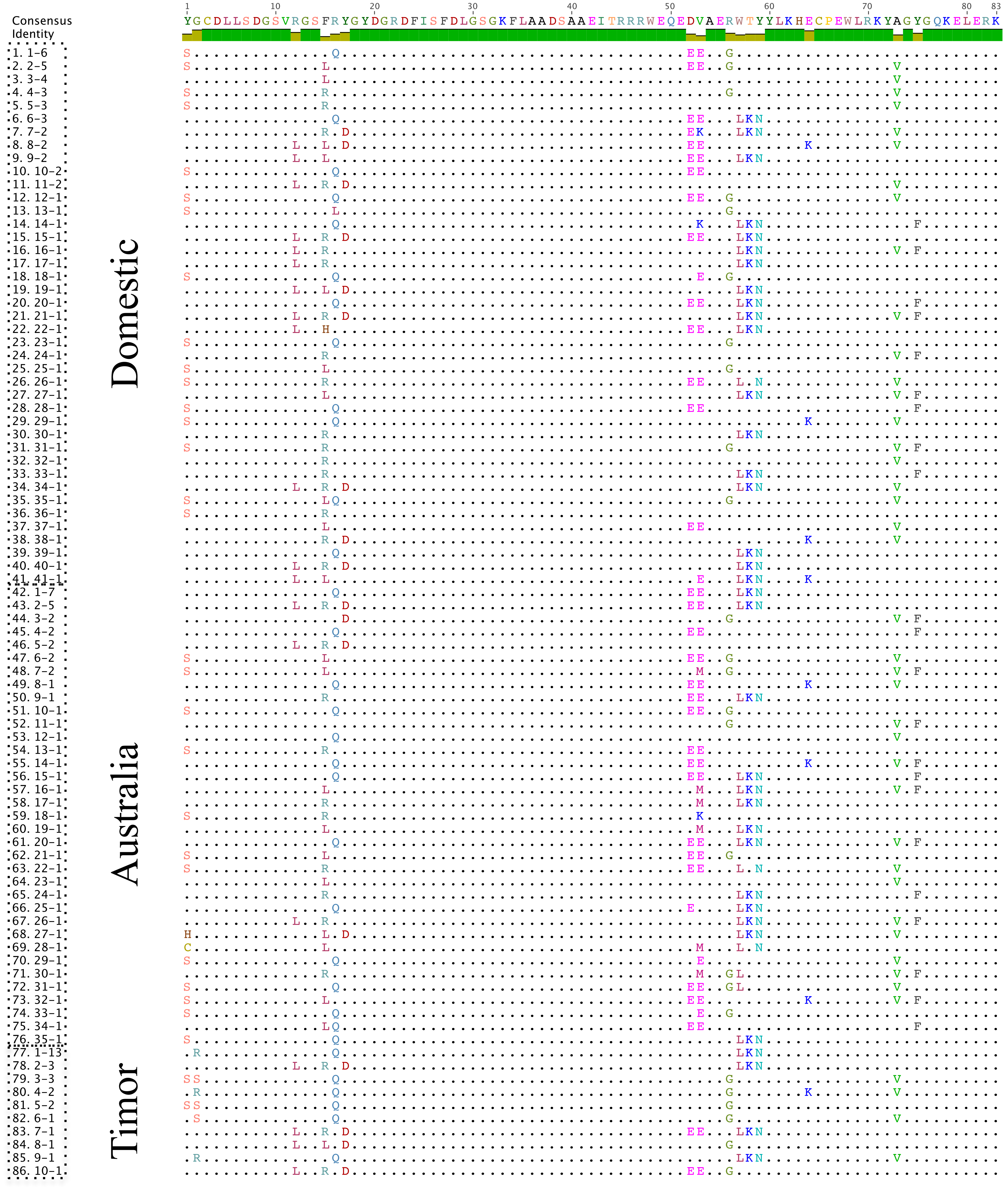

Supplement: Additional file 2: — Amino acid alignment of unique MHC class I sequences from each population. For each unique allele, the first number represents allele ID and the second number represents the total number of individuals in the population carrying the allele. (PNG 1776 kb) [file 12862_2015_546_MOESM2_ESM.png]

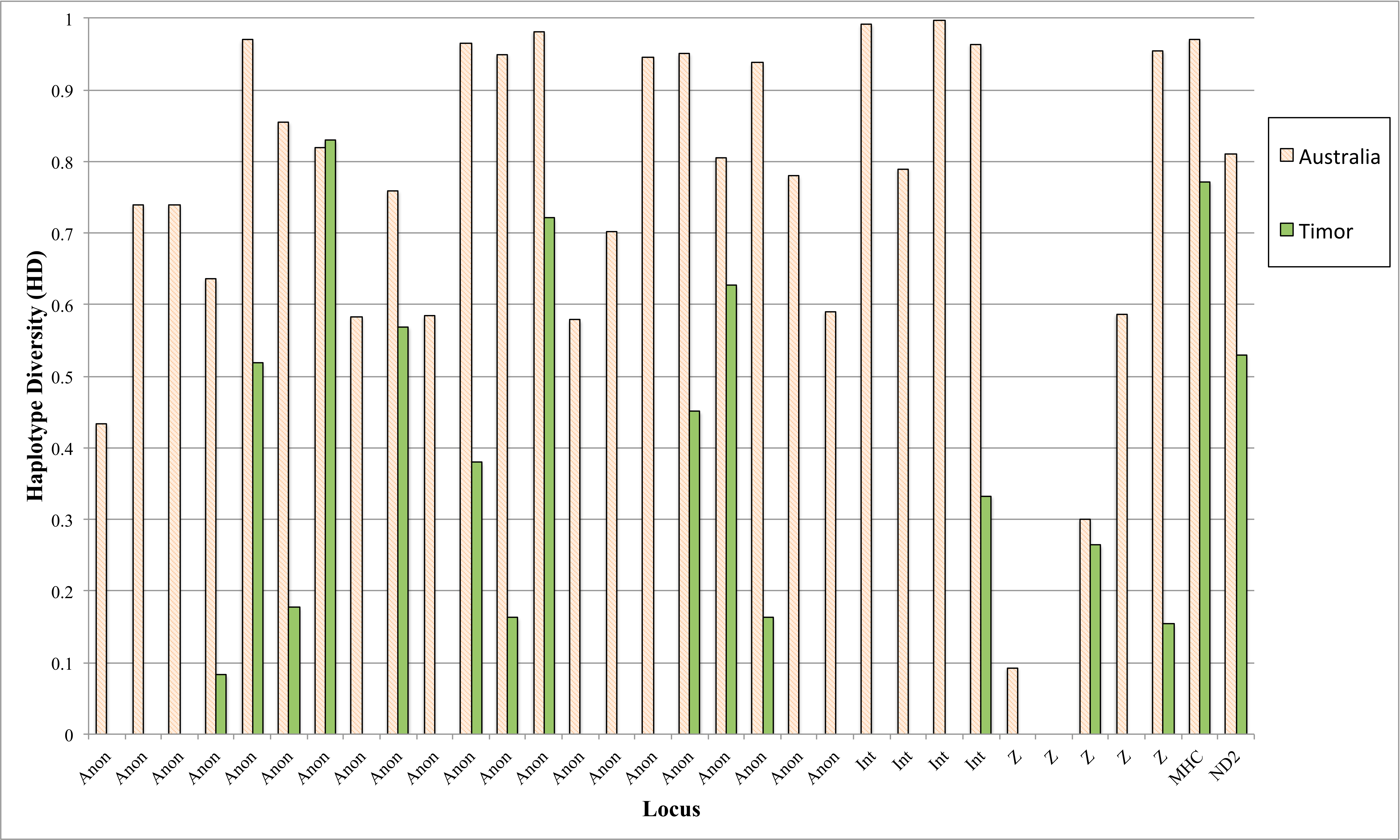

Supplement: Additional file 3: — Island and mainland haplotype diversity (HD) plotted next to each other for all reported loci. Anonymous loci (Anon), introns (Int) and Z linked loci (Z) reported by Balakrishnan & Edwards [37] as well as MHC class I and ND2 from the present study. (PNG 1820 kb) [file 12862_2015_546_MOESM3_ESM.png]

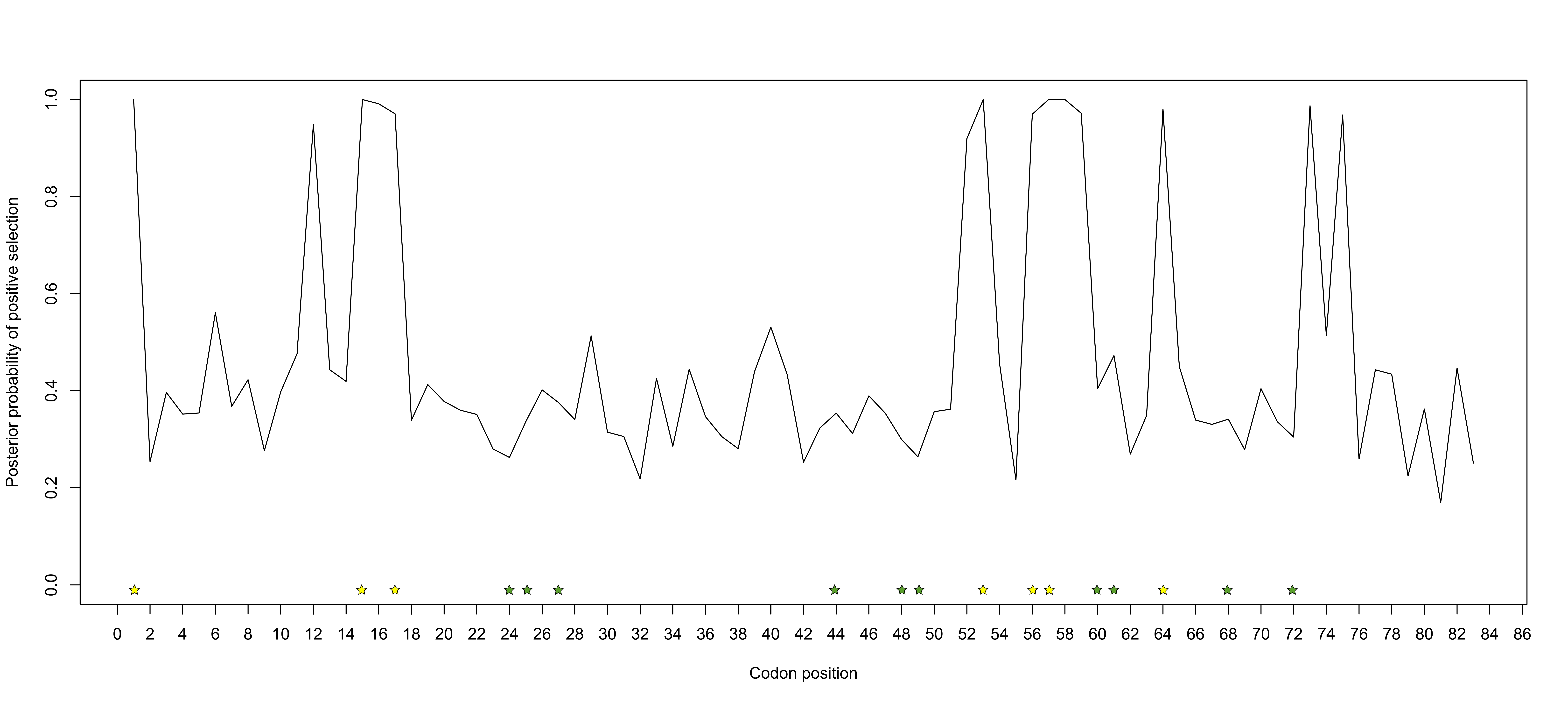

Supplement: Additional file 4: — Output from omegaMap plotting posterior probability of positive selection against codon position for the wild Australian population. Stars represent sites encoding the PBR, with yellow stars indicating ω > 1 and green stars indicating ω < 1. (PNG 773 kb) [file 12862_2015_546_MOESM4_ESM.png]

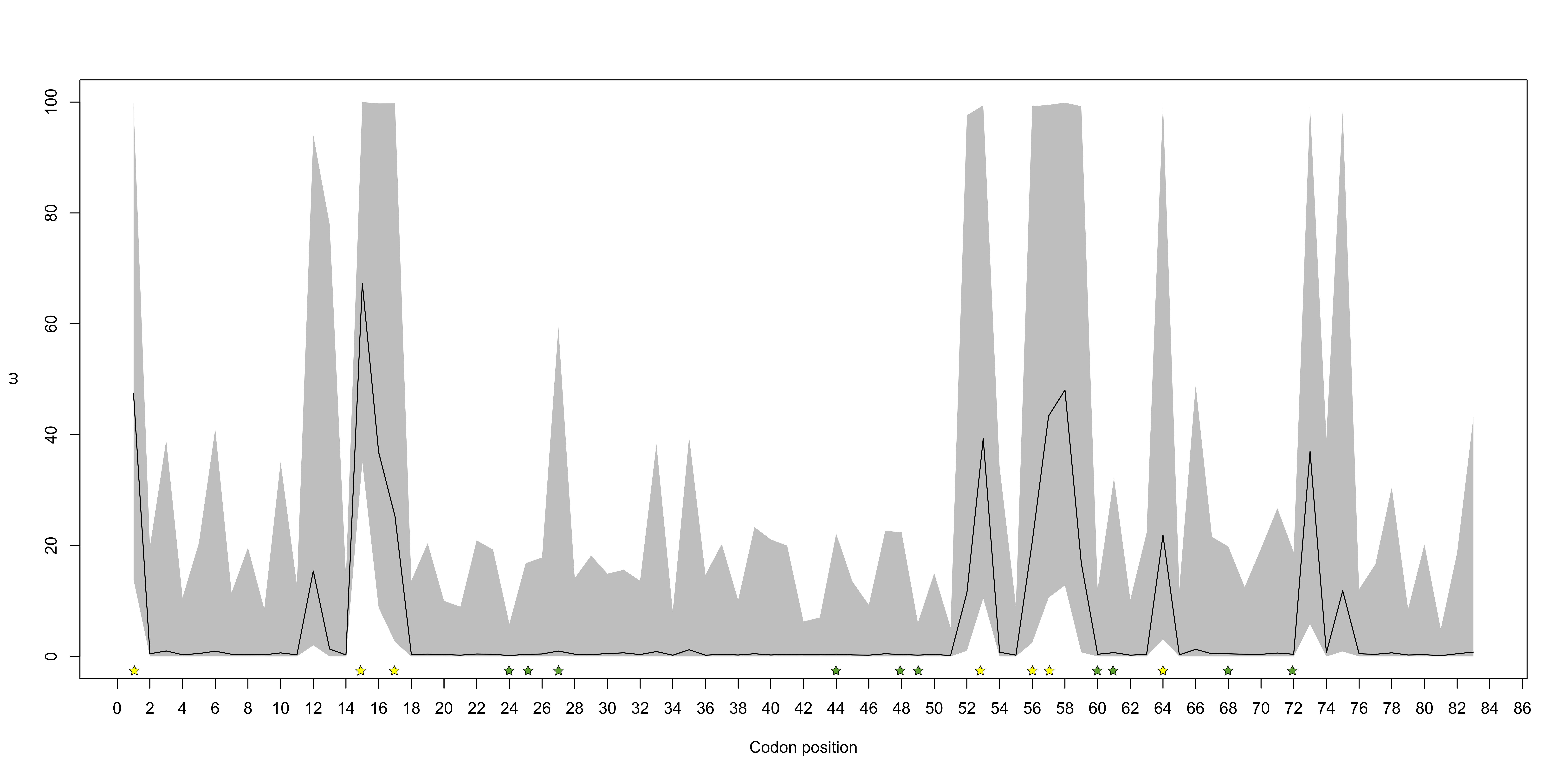

Supplement: Additional file 5: — Output from omegaMap plotting ω against codon position for the domesticated population. The black lines represent omega and the grey shading represents 95 % highest posterior probability densities (HPD). Stars represent sites encoding the PBR, with yellow stars indicating ω > 1 and green stars indicating ω < 1. (PNG 1028 kb) [file 12862_2015_546_MOESM5_ESM.png]

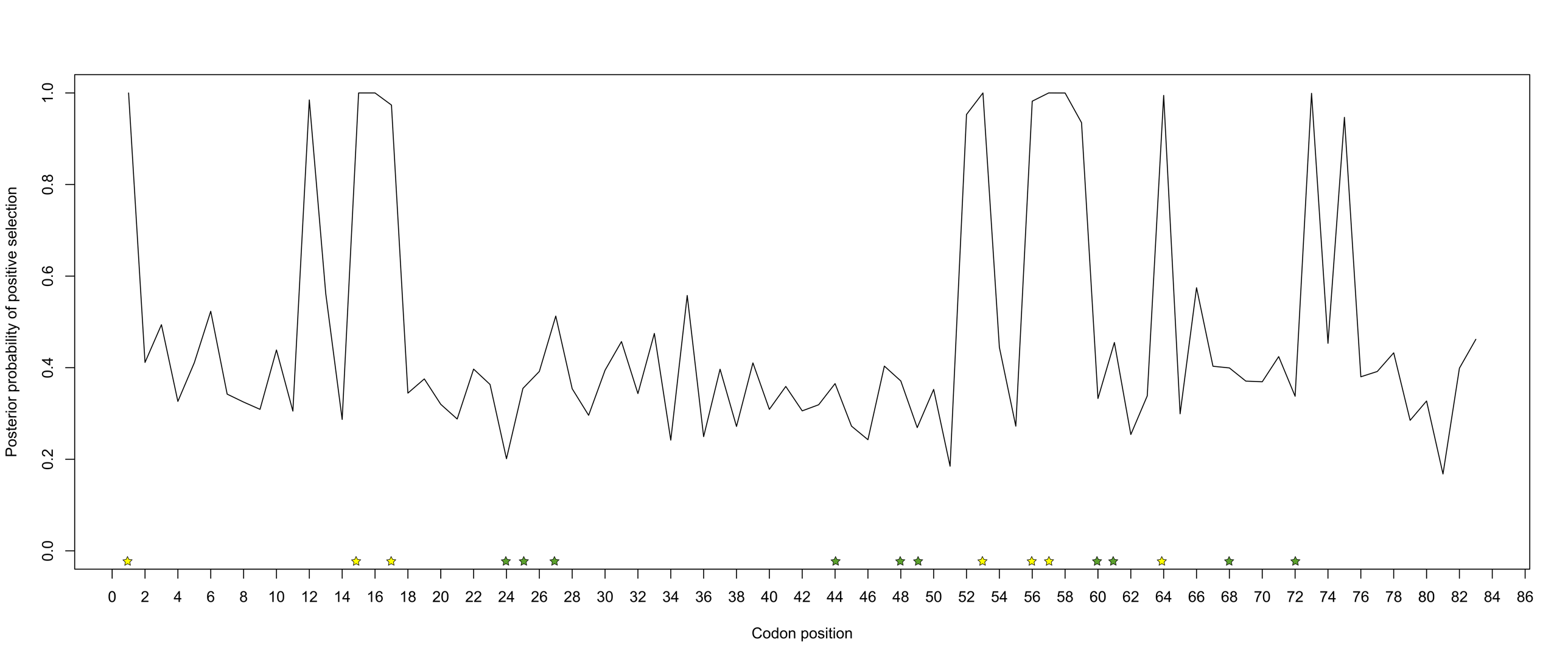

Supplement: Additional file 6: — Output from omegaMap plotting posterior probability of positive selection against codon position for the domesticated population. Stars represent sites encoding the PBR, with yellow stars indicating ω > 1 and green stars indicating ω < 1. (PNG 809 kb) [file 12862_2015_546_MOESM6_ESM.png]

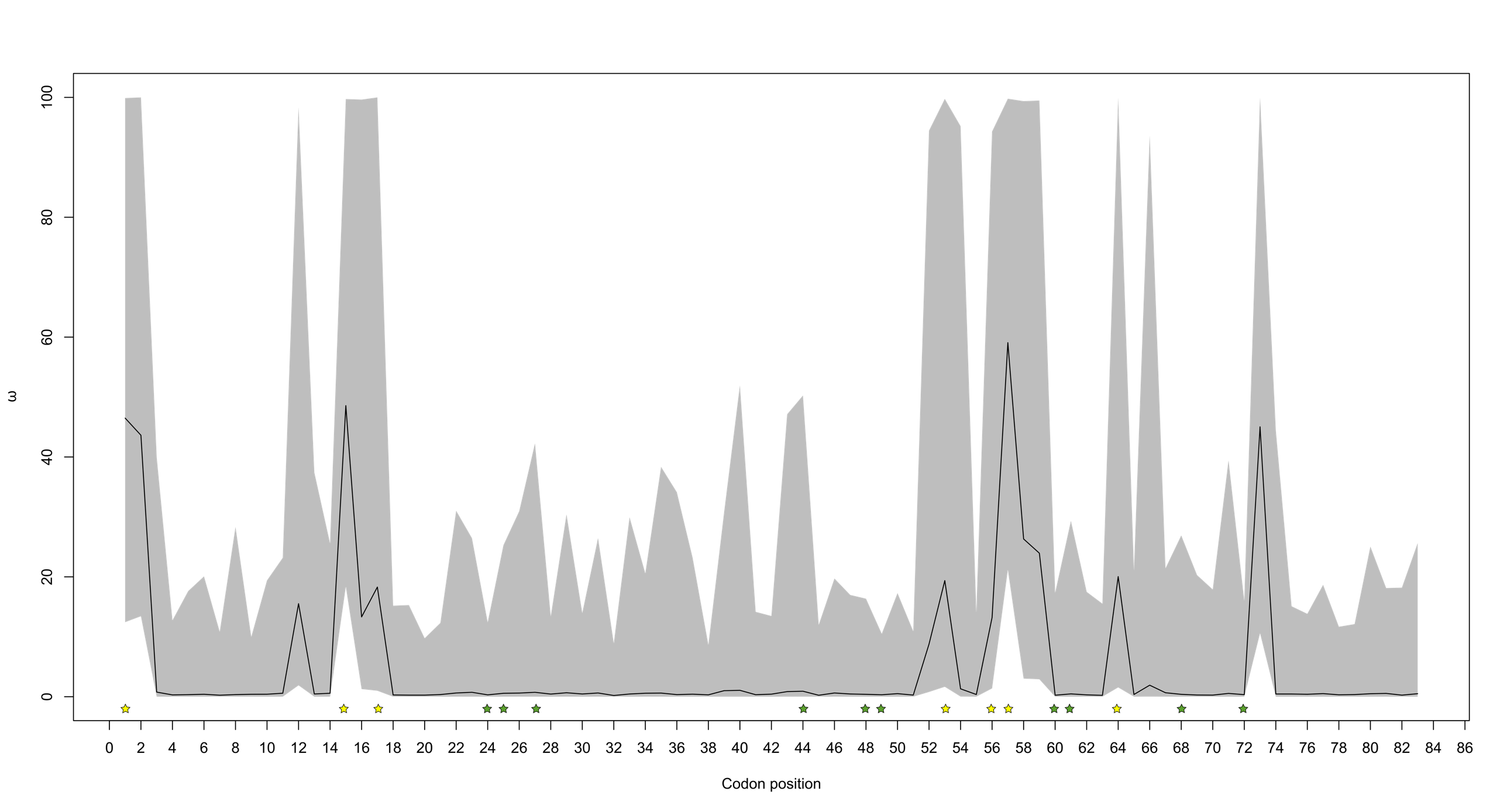

Supplement: Additional file 7: — Output from omegaMap plotting ω against codon position for the Timor population. The black lines represent omega and the grey shading represents 95 % highest posterior probability densities (HPD). Stars represent sites encoding the PBR, with yellow stars indicating ω > 1 and green stars indicating ω < 1. (PNG 1064 kb) [file 12862_2015_546_MOESM7_ESM.png]

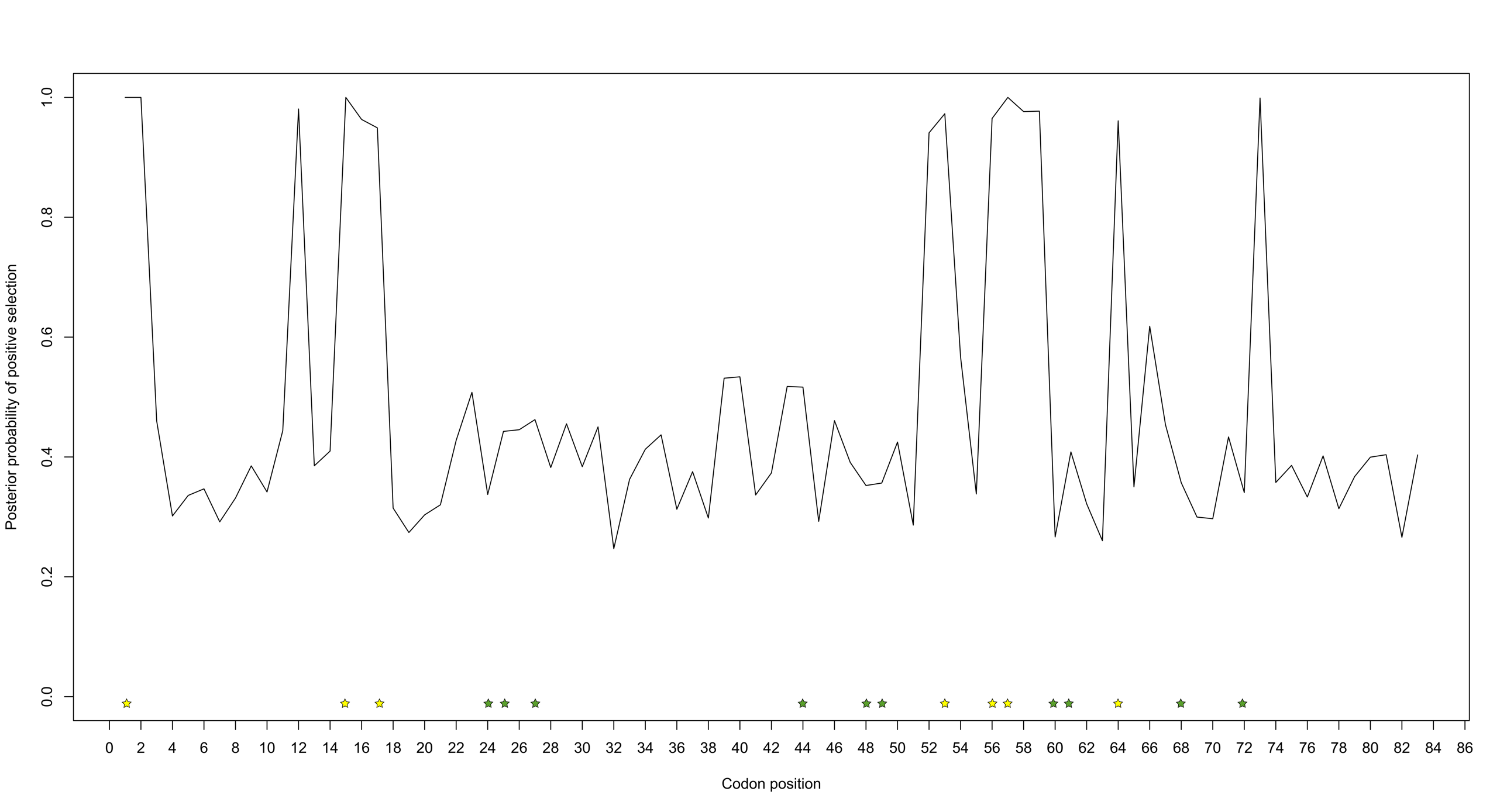

Supplement: Additional file 8: — Output from omegaMap plotting posterior probability of positive selection against codon position for the Timor population. Stars represent sites encoding the PBR, with yellow stars indicating ω > 1 and green stars indicating ω < 1. (PNG 870 kb) [file 12862_2015_546_MOESM8_ESM.png]
