# Supplementary material for: High major histocompatibility complex class I polymorphism despite bottlenecks in wild and domesticated populations of the zebra finch (Taeniopygia guttata)
Source: BMC Evol Biol. 2015 Dec 1;15:265. doi: 10.1186/s12862-015-0546-3 (PMC4667478; doi:10.1186/s12862-015-0546-3)
Supplement: Additional file 9: — BottleSim output showing the final number of alleles (±SE) at 10, 20 and 50 % post-bottleneck growth rate for a bottleneck size of 10 and 100 individuals. (PDF 41 kb) [file 12862_2015_546_MOESM9_ESM.pdf]

	**Bottleneck Size**	
	10	100
Growth Rate	Alleles (±SE)	Alleles (±SE)
10%	4.06 (±0.03)	21.7 (±0.07)
20%	6.45 (±0.04)	27.7 (±0.07)
50%	9.78 (±0.05)	32.4 (±0.05)
